# The α subunit of the heterotrimeric G protein regulates mesophyll CO_2_ conductance and drought tolerance in rice

**DOI:** 10.1111/nph.17730

**Published:** 2021-09-30

**Authors:** Yotam Zait, Ángel Ferrero‐Serrano, Sarah M. Assmann

**Affiliations:** ^1^ Biology Department Penn State University 208 Mueller Laboratory University Park PA 16802 USA

**Keywords:** CO_2_ assimilation, *d1*, drought, heterotrimeric G protein, mesophyll conductance, *Oryza sativa*, stomatal conductance

## Abstract

- Mesophyll conductance *g*_m_ determines CO_2_ diffusion rates from mesophyll intercellular air spaces to the chloroplasts and is an important factor limiting photosynthesis. Increasing *g*_m_ in cultivated plants is a potential strategy to increase photosynthesis and intrinsic water use efficiency (WUE_i_). The anatomy of the leaf and metabolic factors such as aquaporins and carbonic anhydrases have been identified as important determinants of *g*_m_. However, genes involved in the regulation and modulation of *g*_m_ remain largely unknown.
- In this work, we investigated the role of heterotrimeric G proteins in *g*_m_ and drought tolerance in rice *d1* mutants, which harbor a null mutation in the Gα subunit gene, *RGA1*.
- *d1* mutants in both cv Nipponbare and cv Taichung 65 exhibited increased *g*_m_, fostering improvement in photosynthesis, WUE_i_, and drought tolerance compared with wild‐type. The increased surface area of mesophyll cells and chloroplasts exposed to intercellular airspaces and the reduced cell wall and chloroplast thickness in the *d1* mutant are evident contributors to the increase in *g*_m_.
- Our results indicate that manipulation of heterotrimeric G protein signaling has the potential to improve crop WUE_i_ and productivity under drought.

## Introduction

Drought is one of the most important environmental variables affecting crops, causing more annual loss in crop yield than all pathogens combined (Gupta *et al*., 2020). This constraint on global crop production will be further exacerbated by climate change (Wassmann *et al*., 2009). Drought commonly compromises paddy‐grown rice and is the single biggest constraint on yield production in rain‐fed rice (Tuong & Bouman, 2003). Because rice is the staple food for more than half of the world's population, the development of varieties with improved photosynthesis and water (H_2_O) use efficiency in the face of suboptimal conditions is of enormous importance for both present‐day and future food security (Long *et al*., 2015).

During photosynthesis, the provision of CO_2_ to the sites of carboxylation in the chloroplast stroma first requires the diffusion of CO_2_ from the atmosphere through stomata into the leaf intercellular air spaces, and is thus dependent on stomatal conductance *g*_s_. In the next phase, CO_2_ flows from intercellular air spaces to the fixation site through several resistances: cell wall, cell membrane, cytoplasm, chloroplast envelope, and finally the stroma, where CO_2_ encounters Rubisco. These resistances in series determine the mesophyll conductance to CO_2_ *g*_m_, which is calculated as the photosynthetic rate *A*_n_ divided by the CO_2_ drawdown from its concentration in the intercellular air spaces *C*_i_ to the site of CO_2_ fixation *C*_c_. High mesophyll conductance ensures sufficient CO_2_ concentration at the fixation site and is, therefore, strongly correlated with photosynthesis (Harley *et al*., 1992; Gago *et al*., 2020). The intrinsic water use efficiency WUE_i_ (the photosynthesis to stomatal H_2_O conductance ratio) can be improved by limiting transpiration via reducing stomatal conductance to H_2_O vapor *g*_sw_ and/or by increasing photosynthesis (Barbour *et al*., 2010). Enhancing or maintaining *g*_m_ when stomatal conductance to H_2_O decreases is another strategy to improve WUE_i_ (Barbour *et al*., 2010; Tomeo & Rosenthal, 2017).

Two types of components contribute to mesophyll conductance. First, at the structural level, anatomical characteristics, most notably mesophyll surface area exposed to intercellular air spaces (Evans *et al*., 1994), chloroplast distribution in cells (Tholen *et al*., 2008), and cell wall thickness (Terashima *et al*., 2011; Ellsworth *et al*., 2018), impact *g*_m_. Second, at the biochemical level, *g*_m_ may be regulated by cooporins (aquaporins that are permeable to CO_2_), facilitating CO_2_ diffusion through the cell membrane and chloroplast envelope (Evans *et al*., 2009), and by the efficiency of the conversion of CO_2_ to bicarbonate (HCO_3_^−^) by carbonic anhydrases (CAs) (Gillon & Yakir, 2000).

Heterotrimeric G proteins are interesting candidates involved in the regulation of stomatal conductance (Ferrero‐Serrano & Assmann, 2016), yet their potential role in the regulation of *g*_m_ remains unexplored. Heterotrimeric G proteins are GDP‐GTP binding proteins composed of Gα, Gβ, and Gγ subunits that work with G protein‐coupled receptors to transduce extracellular signals into intracellular responses. G proteins impact numerous aspects of plant morphology and modulate responses to many environmental stresses (Wang *et al*., 2001; Fan *et al*., 2008; Zhang *et al*., 2008; Nilson & Assmann, 2010a; Chakravorty *et al*., 2011), including drought stress (Nilson & Assmann, 2010b; Ferrero‐Serrano & Assmann, 2016).

The rice genome contains a single canonical Gα (*RGA1*) and four extra‐large Gα subunits (*XLG*), one Gβ (*RGB1*), and four Gγ (*RGG1*, *RGG2*, *GS3*, and *DEP1*) genes, with a fifth related Gγ gene or pseudogene, *OsGGC2* (Perfus‐Barbeoch *et al*., 2004). Mutations in rice G protein subunits have been found to affect numerous agronomically related phenotypes (Botella, 2012; Cui *et al*., 2020). Multiple *RGA1* mutants have been identified that result in dwarf plants with short and erect leaves with higher Chl content (Oki *et al*., 2009); these ‘*d1*’ mutants exhibit improved drought resistance (Ferrero‐Serrano & Assmann, 2016; Cui *et al*., 2020) and photoprotection (Ferrero‐Serrano *et al*., 2018). Ferrero‐Serrano & Assmann (2016) showed higher rates of photosynthesis during drought in *d1* compared with wild‐type (WT) despite having the same *g*_sw_. We hypothesized that *d1* exhibits drought tolerance due to its ability to maintain high *g*_m_, thereby maintaining photosynthesis and increasing WUE_i_ under drought. We evaluated the mesophyll conductance to CO_2_ and leaf mesophyll anatomy of *d1* mutants under well‐watered and drought stress conditions to explore the role RGA1 plays in the interplay between *g*_m_ and drought tolerance.

## Materials and Methods

### Plant material and growth conditions

We used WT cv Taichung 65 (T65) and cv Nipponbare (NB), and the mutant plants we used consisted of *d1* in the T65 background in which there is a 2 bp deletion in *RGA1* and *d1* in the NB background in which there is a one base substitution in *RGA1* that results in a protein null phenotype based on immunoblot analysis (Oki *et al*., 2009) (*d1‐1* in the T65 background and *d1‐5* in the NB background). Plants were grown two per pot and five pots per genotype for 60 d prior to the initiation of drought. Plants were grown in 10 l pots containing Metro‐mix 360 potting mixture (Sun Gro Horticulture Canada Ltd, Vancouver, Canada), initially irrigated daily to full capacity so that the relative soil H_2_O content (RSWC) never dropped below 85%. Glasshouse temperatures were set to 30°C : 22°C, day : night. Light intensity in the glasshouse during the measurement period averaged *c*. 600 µmol m^−2^ s^−1^. After 60 d from emergence, the soil H_2_O content was brought to 80–90% and the soil was then allowed to dry progressively for 20 d, during which physiological measurements (described in the next section) were taken. During the drought experiment, pots were weighed daily, and RSWC was determined by lysimetry: RSWC (%) = (PW − DW)/(t_0_W − DW) × 100 (PW, pot weight; DW, pot dry weight; t_0_W, pot weight on day 0 of the drought experiment).

### Gas exchange measurements

Gas exchange and fluorescence measurements were conducted using the LI‐6800 photosynthesis system (Li‐Cor Inc., Lincoln, NE, USA) equipped with a 2 cm^2^ fluorescence leaf chamber. Measurements were conducted at light intensities of 1500 μmol m^−2^ s^−1^ (determined as saturating light from *A*_n_–photosynthetic photon flux density (PPFD) curves) and an air flow rate of 300 μmol s^−1^. The block temperature was set to 32°C, and the relative humidity was controlled at 50% using the LI‐6800 humidifier and desiccant. All gas exchange measurements were made between 09:00 h and 13:00 h. We chose the center of the newly expanded leaf, 5–10 cm from the tip of the leaf lamina, for measurement. After a leaf was clamped in the LI‐6800 chamber, we allowed 10 min of acclimation to a fixed reference CO_2_ concentration of 400 μmol mol^−1^ air. Then, CO_2_ response curves for gas exchange combined with Chl fluorescence were measured. The initial CO_2_ concentration was set to ambient CO_2_ concentration (400 μmol mol^−1^ air), which was then reduced to 300, 200, 150, 100, and 50 ppm. Then, the CO_2_ concentration was returned to 400 μmol mol^−1^ air and then increased to 600, 800, 1000, 1200, and 1600 μmol mol^−1^ air. Mesophyll conductance *g*_m_ was estimated using the variable *J* method (Harley *et al*., 1992; Loreto *et al*., 1992):

$$g_{m}=\frac{A_{n}}{C_{i}‐C_{c}}$$

(*A*_n_, net photosynthesis rate; *C*_i_, CO_2_ concentration in the leaf intercellular airspaces; *C*_c_, CO_2_ concentration in the chloroplast stroma); *A*_n_ and *C*_i_ were both determined from gas exchange measurements, and *C*_c_ is estimated from combined measurements of gas exchange and Chl fluorescence as:

$$C_{c}=\frac{\Gamma^{*}\left[J+8\left(A_{N}+R_{d}\right)\right]}{J‐4(A_{N}+R_{d})}$$

(*J*, electron‐transport rate, calculated based on Chl fluorescence; *R*_d_, nonphotorespiratory respiration in light; $\Gamma^{*}$, apparent photo‐compensation point, estimated according to Laisk & Loreto (1996)). CO_2_ response curves were measured under three different light intensities (100, 200, and 400 µmol m^−2^ s^−1^) and at 21% oxygen (O_2_) and fitted linearly. The *y* and *x* values of the average of three CO_2_ response intersections were taken as *R*_d_ and $C_{i}^{*}$ (Supporting Information Fig. S1).

The $\Gamma^{*}$ is dependent on *g*_m_ and *R*_d_ as follows (von Caemmerer *et al*., 1994):

$\Gamma^{*}=C_{i}^{*}+\frac{R_{d}}{g_{m}}$

However, since no *g*_m_ value was available before the measurements, the conversion was not completed and the $C_{i}^{*}$ was taken as a representation of $\Gamma^{*}$ (Qiu *et al*., 2017). We have also tested constant $\Gamma^{*}$ values obtained from Bernacchi *et al*. (2002) (Fig. S2).

The electron transport rate *J* is calculated as follows:

$$J=\Phi_{\text{PSII}}\times \text{PPFD}\times \alpha \times \beta$$

(*α*, coefficient of leaf absorbance; *β*, fraction of absorbed quanta that reaches photosystem II (PSII)). The quantum yield of Φ_PSII_ was calculated according to Genty *et al*. (1989):

$$\Phi_{\text{PSII}}=\frac{F_{m}^{\prime }‐F_{t}}{F_{m}^{\prime }}$$

(*F*_t_, steady‐state fluorescence; $F_{m}^{\prime }$, maximal fluorescence in the light‐adapted state, after a light‐saturating pulse of the multiphase flash fluorometer of the LI‐6800). The quantum yield of CO_2_ fixation was defined as:

$$\Phi_{{\text{CO}}_{2}}=\frac{A_{n}+R_{d}}{\text{PPFD}}$$

The combined use of gas exchange and Chl fluorescence relies on the assumption that there is a linear relationship between $\Phi_{\text{PSII}}$ and $\Phi_{{\text{CO}}_{2}}$ under nonphotorespiratory conditions, since CO_2_ fixation is the only sink for electrons (Valentini *et al*., 1995). From the response curve measured at 1% O_2_, we performed a linear regression of actual quantum efficiency of PSII Φ_PSII_ and the quantum yield of CO_2_ fixation $\Phi_{{\text{CO}}_{2}}$ to obtain a regression coefficient *k*. The theoretical model and experimental observations were limited to the linear region of $\Phi_{{\text{CO}}_{2}}<0.05$ and $\Phi_{\text{PSII}}<0.5$ (Fig. S3). The slopes of the linear regression *k* and the *y*‐axis intercept *b* were used to recalculate the *α* × *β* for the electron‐transport rate *J* based on Chl fluorescence measured at 21% O_2_ as:

$$J_{\text{cal}}=\frac{4(\Phi_{\text{PSII}}‐b)}{k}\times \text{PPFD}$$

The *C*_i_ values were corrected for cuticular diffusivities to H_2_O and CO_2_ according to Boyer *et al*. (1997):

$$C_{i}=C_{a}‐1.6\frac{A_{n}}{E_{l}‐2E_{c}}\left(W_{i}‐W_{a}\right)$$

(*C*_a_, CO_2_ concentration of the air surrounding the leaf (taken as the Li‐Cor CO_2_ reference); *W*_i_ and *W*_a_, mole fractions of H_2_O in the leaf intercellular air spaces and in the air surrounding the leaf, respectively; *E*_l_, leaf transpiration; *E*_c_, cuticular transpiration (assumed to be constant at 5 mmol H_2_O m^–2^ s^–1^) (Flexas & Medrano, 2002).

CO_2_ concentration in the chloroplast stroma *C*_c_ was then calculated with the observed *g*_m_ value according to Fick's first law of diffusion:

$$C_{c}=C_{i}‐\frac{A_{n}}{g_{m}}$$

The *A*_n_–*C*_i_ curves were converted into *A*_n_–*C*_c_ curves and analyzed using the plantecophys R package for analyzing and modeling leaf gas exchange data (Duursma, 2015). The maximum carboxylation rate *V*_cmax_ and rate of electron transport *J*_max_ were computed.

### Temperature dependence of photosynthesis and *g*_m_

Leaf temperatures were controlled with the LI‐6800 thermocouple by fixing the block temperature to the desired temperature. Well‐watered plants were initially measured at 20°C and then at 25, 30, 35, and 40°C. We allowed at least 30 min at each temperature after the leaf temperature had stabilized before measurements were taken. After *A*_n_ and *g*_sw_ reached steady state, a new *A*_n_–*C*_i_ curve was performed as described earlier herein.

### Leaf anatomy

At the conclusion of the gas exchange measurements, leaf samples from T65 WT, and *d1* plants were cut from the exact leaf patches that were measured for physiology and immediately fixed in a buffer containing 2.8% glutaraldehyde in 0.1 M HEPES buffer, pH 7.2 (with 0.02% v/v Triton X‐100) at room temperature for 2 h, and then overnight at 4°C. Samples were then postfixed in 1% osmium tetroxide overnight in a lightproof container and then dehydrated in a graded acetone series (20–100%). The dehydrated segments were subsequently embedded in Spurr's resin and polymerized at 60°C for 2 d. Transverse sections, 1000 µm thick, were prepare using an ultramicrotome (Leica EM UC6; Leica Mikrosysteme GmbH, Vienna, Austria), stained with 1% toluidine blue in 1% borax (pH 9.0) and observed under a light microscope (Olympus BX51; Olympus, Tokyo, Japan). Ultrathin cross‐sections for transmission electron microscopy (TEM) were cut with glass knives on an ultramicrotome and the sections were stained with uranyl acetate and lead citrate before viewing with an FEI Tecnai G2 Spirit BioTwin (FEI; Hillsboro, OR, USA). Sections were imaged and the mesophyll surface area exposed to intercellular air spaces *S*_m_ and the chloroplast surface area exposed to intercellular air spaces *S*_c_ were measured using ImageJ software (https://imagej.nih.gov/ij/; US National Institute of Health, Bethesda, MD, USA), and calculated as described in Evans *et al*. (1994) and Syvertsen *et al*. (1995): S=(l/W)×1.42 (Scafaro *et al*., 2011; *S* is *S*_m_ or *S*_c_; *l*, total perimeter of mesophyll cells or chloroplasts facing the intercellular air space; *W*, the analyzed cross‐section width; 1.42, curvature correction factor for the ellipsoidal mesophyll cell‐shape (Evans *et al*., 1994)). The volume fraction of intercellular air space was calculated from the optical cross‐sections as $f_{\text{ias}}=S_{\text{ias}}/\left(S_{m}+S_{\text{ias}}\right)$ (Xiong *et al*., 2016; *S*_ias_, cross‐sectional area of the intercellular air space measured with ImageJ). Mesophyll conductance modeled with the anatomic parameters was calculated according to the one‐dimensional model of Tomás *et al*. (2013):

$$g_{m}=\frac{1}{\frac{1}{g_{\text{ias}}}+\frac{RT_{k}}{Hg_{\text{liq}}}}$$

(*g*_ias_, CO_2_ conductance in the gas phase from the substomatal cavities to the outer surface of the cell wall surface; *g*_liq_, CO_2_ liquid‐phase conductance from the outer surface of cell wall surface to the chloroplasts; *R*, gas constant (Pa m^3^ mol^−1^ K^−1^); *T*_k_, absolute temperature (kelvin); *H*, Henry's law constant (atm m^3^ mol^−1^)). The gas‐phase conductance to CO_2_ was calculated as:

$$g_{\text{ias}}=\frac{D_{a}f_{\text{ias}}}{\Delta L_{\text{ias}}\tau }$$

(*D*_a_, diffusivity of CO_2_ in air at 25°C (0.000151 m^2^ s^−1^); *f*_ias_, fraction of leaf air space; Δ*L*_ias_, effective diffusion path length in the gas phase, estimated as half the mesophyll thickness; *τ*, tortuosity of the diffusion path (1.57 mm^−1^); Tomás *et al*., 2013). The CO_2_ liquid‐phase diffusion conductance through cell wall (*g*_cw_), cytosol (*g*_cyt_), and stroma (*g*_st_) were calculated according to a general formula:

$$g_{i}=\frac{D_{w}r_{\text{f,}i}p_{i}}{\Delta L_{i}}$$

(*g_i_* (*i* = cw, cyt, or st), individual component of the liquid‐phase CO_2_ conductance; *D*_w_, CO_2_ aqueous diffusion coefficient at 25°C (1.79 × 10^–9^ m^2^ s^−1^); *p_i_*, effective porosity (1 m^3^ m^−3^ for cytosol and stroma; for the cell wall we assumed identical *p_i_* for the *d1* and WT of 0.3 m^3^ m^−3^); *r*_f,_ *_i_*, factor accounting for the reduction in diffusion due to the presence of solutes and macromolecules (dimensionless, 1.0 for cell wall and 0.3 for cytosol and stroma; Niinemets & Reichstein, 2003); Δ*L_i_*, diffusion path length (meters) for each component along the CO_2_ diffusion pathway). Next, the liquid‐phase conductance *g*_liq_ was calculated as the sum of the inverse of the individual component's conductances, in series:

$$g_{\text{liq}}=\frac{S_{m}}{\frac{1}{g_{\text{cw}}}+\frac{1}{g_{\text{pl}}}+\frac{1}{g_{\text{cyt}}}+\frac{1}{g_{\text{env}}}+\frac{1}{g_{\text{st}}}}S$$

The plasma membrane conductance *g*_pl_ and the chloroplast envelope conductance *g*_env_ were each considered to be 0.0035 m s^−1^, as described in Evans *et al*. (1994).

### Transcriptome analysis

We reanalyzed RNA‐sequencing (RNA‐seq) data from a previously published study of 2‐month‐old WT and *d1* T65 plants that were grown under well‐watered conditions equivalent to those described here (Ferrero‐Serrano *et al*., 2018). These data are publicly available in the Gene Expression Omnibus (GSE103747). The data analyzed were originally collected from the flag leaf at the region of maximum width; that is, the same region as sampled in this study for gas exchange and leaf anatomy. Statistically different expression levels between T65 and *d1* were considered to occur when *q* < 0.05 and fragments per kilobase of exon per million mapped fragments FPKM > 1 in at least one genotype.

## Results

We compared the physiological performance of rice WT and *d1* mutants in the T65 and NB cultivars under well‐watered and water‐limited conditions, with the goal of testing whether the ability to tolerate drought in *d1* (Ferrero‐Serrano & Assmann, 2016) is associated with higher mesophyll conductance *g*_m_ and WUE_i_.

**Fig. 1:**
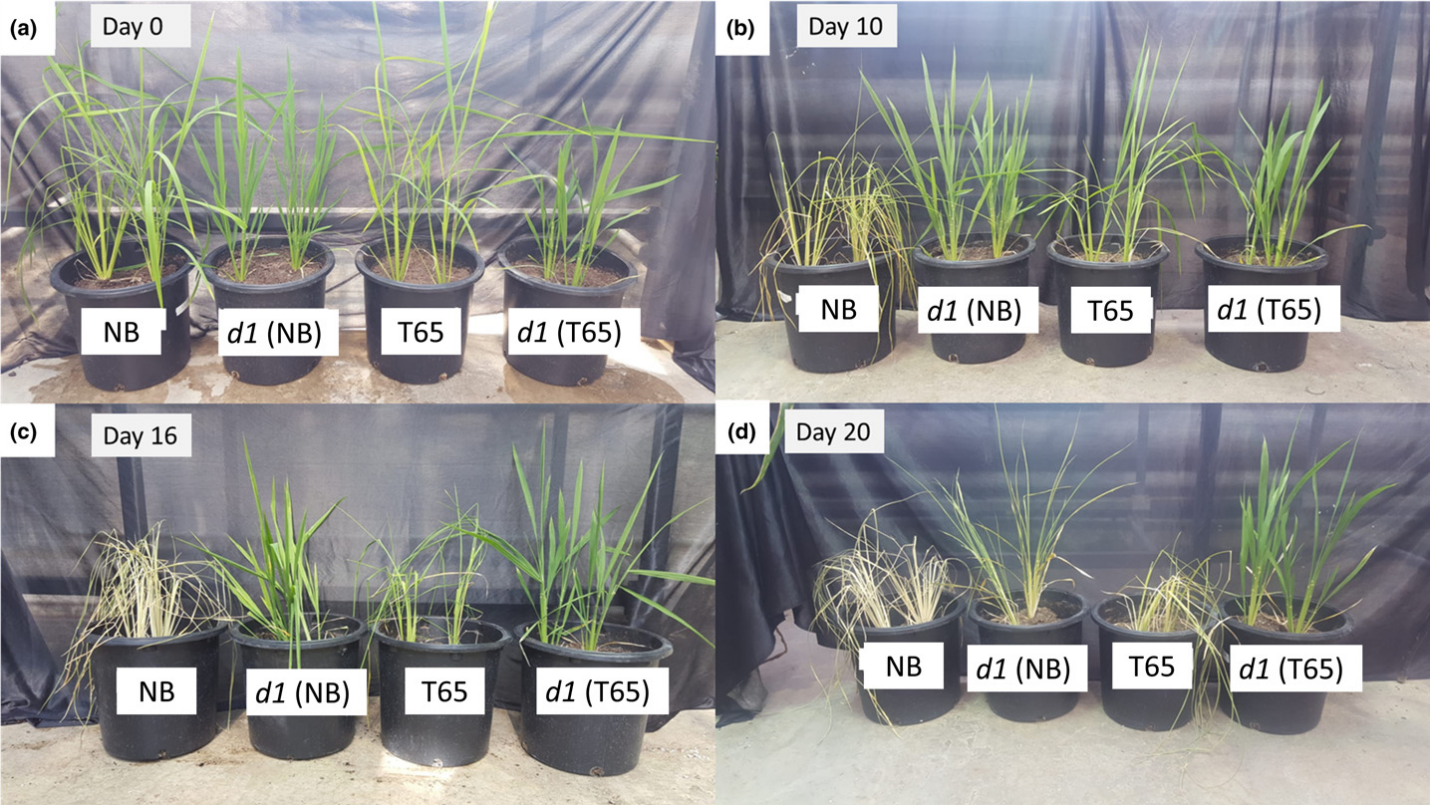
Rice *d1* mutants in both ‘Nipponbare’ (NB) and ‘Taichung 65’ (T65) rice cultivars display improved drought tolerance compared with wild‐type. Images of representative wild‐type cultivars, T65 and NB, and corresponding *d1* mutants taken under (a) well‐watered conditions (day 0), (b) 10 d, (c) 16 d, and (d) 20 d after water was withheld.

Fig. 1 shows the order of plant wilting from the beginning of the drought experiment (day 0), indicating higher drought sensitivity for the NB genotypes than the T65 genotypes. NB plants wilted after 10 d, T65 after 16 d, *d1* (NB) after 20 d, and *d1* (T65) was the most drought tolerant and wilted only after 24 d.

**Fig. 2:**
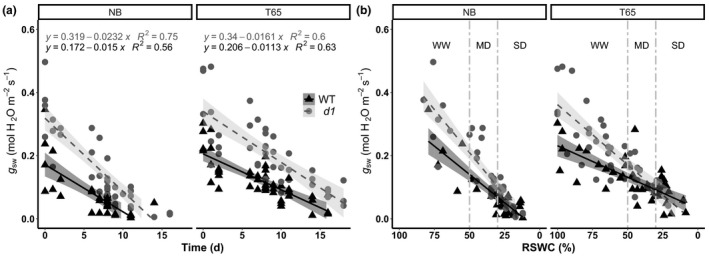
Rice *d1* mutants exhibit greater stomatal conductance than wild‐type (WT) under well‐watered but not drought conditions. Relationships between stomatal conductance to water vapor *g*_sw_ over (a) the drought period and (b) the decline in relative soil water content (RSWC). Vertical lines demarcate the stages corresponding to well‐watered (WW, 100% > RSWC > 50%), moderate drought (MD, 50% > RSWC > 35%), and severe drought (SD, RSWC < 35%). Points are individual measurements. Continuous lines represent the linear fit for the data of the WT rice genotypes, and dashed lines represent the linear fit for the data of the *d1* mutants. The shaded areas show the 95% confidence interval of the regression lines. NB, ‘Nipponbare’; T65, ‘Taichung 65’.

For both WT genotypes and their *d1* respective mutants, the initial *g*_sw_ value was significantly higher in the *d1* mutants (Fig. 2a). The *g*_sw_ values reached nearly zero (i.e. full stomatal closure) after 12 d in WT and 15 d in *d1* in the NB genotype, and after 15 d in WT and 20 d in the *d1* mutant in the T65 genotype. By the time of zero *g*_sw_, leaves were starting to show signs of wilting in the WT plants, but the *d1* mutant leaves remained green and turgid in both genotypic backgrounds. Accordingly, drought stress levels were defined not by days since watering was withheld but by three intervals of RSWC (Fig. 2b), with well‐watered defined as 100% > RSWC > 50%, moderate drought as 50% > RSWC > 35%, and severe drought as RSWC < 35%.

**Fig. 3:**
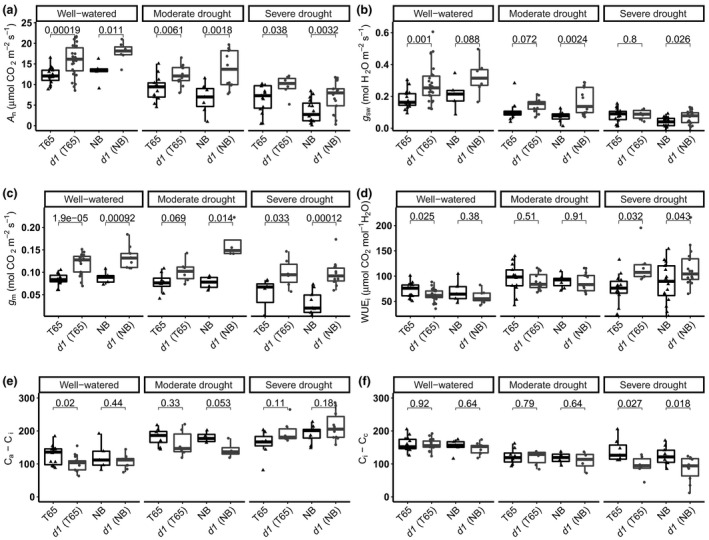
Rice *d1* mutants exhibit improved CO_2_ assimilation rate and mesophyll conductance under well‐watered and drought conditions compared with wild‐type (WT). (a–f) Boxplots of physiological parameters of the different rice cultivars: WT ‘Taichung 65’ (T65) and ‘Nipponbare’ (NB), and *d1* in the T65 background ‘*d1* (T65)’ and *d1* in the NB background ‘*d1* (NB)’, under well‐watered conditions (relative soil water content (RSWC) 100–50%), moderate drought stress (RSWC 50–35%) and severe drought stress (RSWC < 35%), for (a) CO_2_ assimilation *A*_n_, (b) stomatal conductance to water vapor *g*_sw_, (c) mesophyll conductance to CO_2_ *g*_m_, (d) leaf intrinsic water use efficiency WUE_i_, (e) the air to intercellular airspace CO_2_ drawdown (*C*_a_ − *C*_i_), and (f) the intercellular airspace to mesophyll CO_2_ drawdown (*C*_i_ − *C*_c_). The upper and lower parts of the boxes represent the 25^th^ and 75^th^ percentiles, respectively. The horizontal line within the boxes marks the median. The whiskers reach the largest and smallest values. Points are individual measurements. Connecting group lines indicate the *P*‐value score of the means between WT and *d1* according to Student's *t*‐test.

Under well‐watered conditions, all gas exchange parameters quantified for the *d1* mutants were greater than for the corresponding WT, and differences were larger in NB than in T65. The photosynthetic rate was significantly higher in the *d1* mutants than in T65 and NB WT (Fig. 3a). The higher photosynthetic rate observed in *d1* under these conditions can be explained by its significantly higher *g*_s_ (Fig. 3b) and, as we hypothesized, significantly higher *g*_m_ (Fig. 3c). It is important to note here that the higher *g*_sw_ leads to slight but statistically significant lower intrinsic leaf‐level water use efficiency in *d1* vs WT under well‐watered conditions (Fig. 3d). The photosynthetic rates in *d1* plants decreased more gradually and were significantly higher than in WT under moderate stress (Fig. 3a). Under severe water stress, the photosynthetic rate was almost twice as high in *d1* than in WT. With regard to *g*_sw_, under severe drought conditions, there was no significant difference in *g*_sw_ between *d1* mutant and WT in either genetic background (Fig. 3b). A higher photosynthetic rate with the same *g*_sw_ led to 50% and 33*%* greater WUE_i_ for the *d1* plants compared with WT T65 and NB, respectively (Fig. 3d). The *d1* mutants in both genetic backgrounds have higher *g*_m_ relative to the corresponding WT under both moderate and severe water stress (Fig. 3c). In both genotypic backgrounds, the *g*_m_ of the *d1* was 30–45% higher than in WT, under well‐watered conditions and throughout the drought time course.

**Fig. 4:**
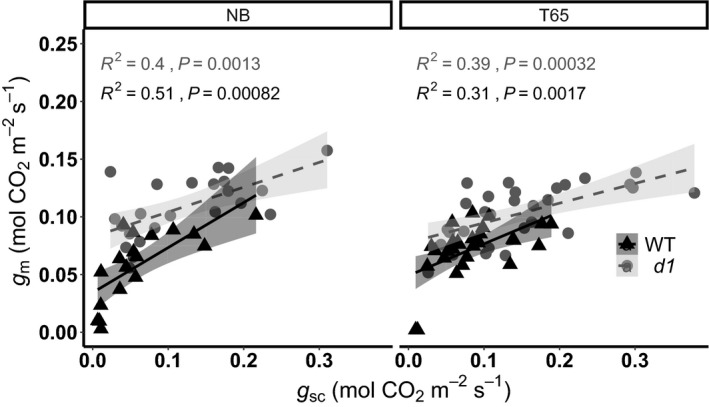
Rice *d1* mutants show a lower decrease in mesophyll conductance *g*_m_ for a given decrease in stomatal conductance *g*_sc_ compared with wild‐type (WT). Relationship between *g*_m_ and *g*_sc_ of the different rice cultivars: WT ‘Taichung 65’ (T65) and ‘Nipponbare’ (NB), and *d1* in T65 background ‘*d1* (T65)’ and *d1* in NB background ‘*d1* (NB)’. Lines represent the linear fit for the data of the different rice genotypes (*n* = 5). The shaded areas show the 95% confidence interval of the regression lines.

Plotting *g*_m_ as a function of stomatal conductance to CO_2_ *g*_sc_ (*g*_sw_/1.6) interestingly shows a steeper decrease in *g*_m_ for a given decrease in *g*_sc_ in the WT plants compared with the *d1* mutants in both T65 and NB backgrounds (Fig. 4).

### Temperature dependence of photosynthesis and *g*_m_

**Fig. 5:**
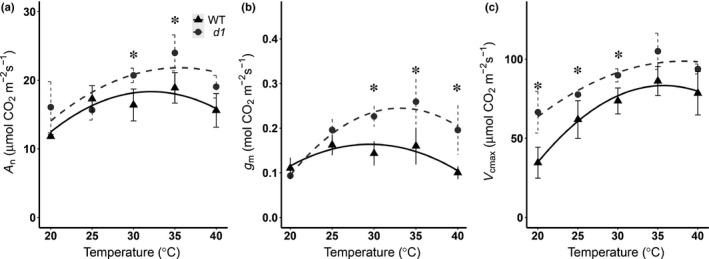
Rice *d1* mutant and wild‐type (WT) exhibit differential temperature dependence of CO_2_ assimilation *A*_n_, mesophyll conductance to CO_2_ *g*_m_, and maximum capacity for Rubisco carboxylation *V*_cmax_. Temperature dependence of (a) *A*_n_, (b) *g*_m_, and (c) *V*_cmax_ in the WT ‘Taichung 65’ and corresponding *d1* mutant. Data are means ± SEs of WT (triangles) and *d1* (circles) (*n* = 4). Asterisks indicate a significant difference between genotypes revealed by Student's *t*‐test (*P* < 0.05). Lines represent cubic curve fit for the data of the different rice genotypes.

We evaluated the effect of leaf temperature on the CO_2_ assimilation *A*_n_, Rubisco capacity for carboxylation *V*_cmax_, and mesophyll conductance *g*_m_ (Fig. 5). The thermal optimum for CO_2_ assimilation rate *V*_cmax_ and mesophyll conductance was 30–35°C for both WT and *d1* (Fig. 5a–c). The CO_2_ assimilation was significantly higher in *d1* than in WT in the temperature range 30–35°C and was not significantly different under lower (20–25°C) and higher (40°C) leaf temperatures (Fig. 5a). The mesophyll conductance was significantly higher in *d1* than in WT in the temperature range 30–40°C and was not significantly different under lower (20–25°C) leaf temperatures (Fig. 5b). The *V*_cmax_ increased with temperature and was higher in the *d1* mutant than in WT in the temperature range 25–30°C (Fig. 5c).

### Relationship between mesophyll conductance and leaf anatomical traits

**Fig. 6:**
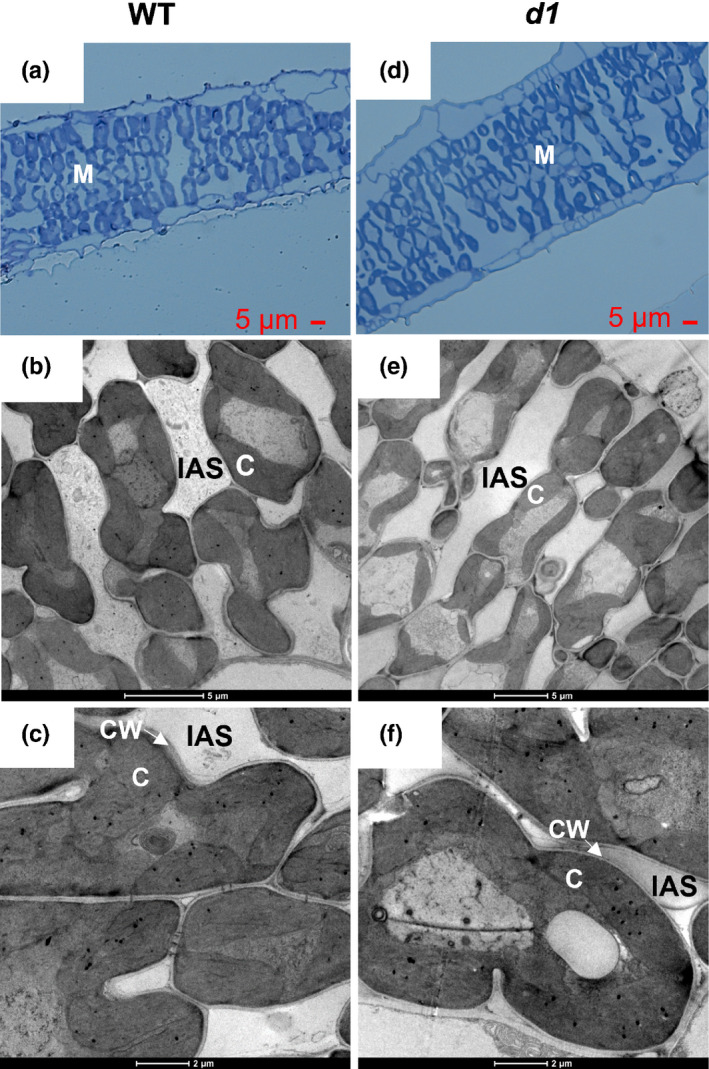
Rice *d1* mutants and wild‐type (WT) display different leaf anatomy. Light micrographs and transmission electron microscope images of (a–c) WT and (d–f) *d1* leaves, showing mesophyll cells (M), the intercellular air space (IAS), cell wall (CW), and chloroplast (C).

**Fig. 7:**
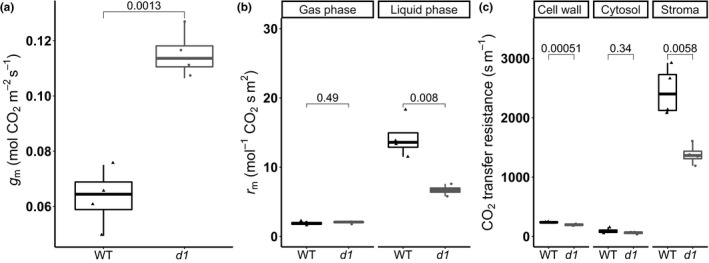
Rice *d1* mutants’ anatomical properties are associated with a reduction in CO_2_ liquid‐phase resistance compared with wild‐type (WT). (a) Mesophyll conductance *g*_m_ modeled with anatomical parameters of the ‘Taichung 65’ (T65) and *d1* mutant. (b) The overall resistance of CO_2_ diffusion imposed by the intercellular air spaces in the gas phase and by the CO_2_ diffusion in the liquid phase in the T65 cultivar and corresponding *d1* mutant. (c) The CO_2_ transfer resistance of the cell wall (*r*_cw_), cytosol (*r*_cyt_) and the chloroplast stoma (*r*_st_) of the T65 cultivar and the corresponding *d1* mutant (c). The upper and lower parts of the boxes represent the 25^th^ and 75^th^ percentiles, respectively. The horizontal line within the boxes marks the median. The whiskers reach the largest and smallest values. Points are individual measurements. Connecting group lines indicate the *P*‐value score of the means between WT and *d1* according to Student's *t*‐test.

The leaf thickness *T*_leaf_, the mesophyll surface area exposed to intercellular air space per unit leaf area *S*_m_, the mesophyll cell surface area occupied by chloroplasts exposed to intercellular air space per unit leaf area *S*_c_, the mesophyll cell wall thickness *T*_cw_, and the chloroplast thickness *T*_chl_ were significantly different between WT and *d1* (Fig. 6; Table 1). Modeling of the diffusion resistance along the CO_2_ diffusion path from intercellular air spaces to the chloroplast stroma indicated significant differences between WT and the *d1* mutant in *g*_m_ (Fig. 7a). Accordingly, the higher *g*_m_ in *d1* mutants compared with WT can be explained at least in part by the anatomy of the mesophyll. The resistance of the liquid phase, requiring diffusion across the plasma membrane, cytosol, chloroplast membranes, and chloroplast stroma, was 53% lower in the *d1* mutants than in WT (Fig. 7b). The cell wall resistance and stroma resistance were 18% and 44% lower, respectively, in *d1* than in WT, whereas the cytosolic resistance was statistically similar (Fig. 7c).

**Table 1:** Structural and anatomical traits of well‐watered rice ‘Taichung 65’ (T65) and the *d1* mutants.

	*T*_leaf_ (μm)	LMA (g m^−^^2^)	*S*_m_ (m^2^ m^−2^)	*S*_c_ (m^2^ m^−2^)	*T*_cw_ (µm)	*T*_cyt_ (µm)	*L*_chl_ (µm)	*T*_chl_ (µm)
T65	68.5 ± 1.7 a	62.9 ± 3.9 a	17.5 ± 0.8 a	13.9 ± 0.7 a	0.128 ± 0.004 a	0.05 ± 0.0013 a	4.3 ± 0.4 a	2.6 ± 0.25b a
*d1*	74.9 ± 2.8 b	66.5 ± 2.7 a	22.5 ± 1.3 b	19.5 ± 1.6 b	0.106 ± 0.004 b	0.03 ± 0.007 a	4.6 ± 0.4 a	1.48 ± 0. 26 b
*P* value	0.016	0.4	0.004	0.004	0.00433	0.216	0.55	0.0084

*T*_leaf_, leaf thickness; LMA, leaf mass per area; *S*_m_, mesophyll surface area exposed to intercellular air space per unit leaf area; *S*_c_, mesophyll cell surface area occupied by chloroplasts exposed to intercellular air space per unit leaf area; *T*_cw_, mesophyll cell wall thickness; *T*_cyt_, distance between cell wall surface and chloroplasts; *L*_chl_, chloroplast length; *T*_chl_, chloroplast thickness.

Values are mean ± SE, *n* = 4 plants per genotype; four to seven images analyzed per plant. Letters indicate statistical difference according to Student's *t*‐test.

### *A*_n_–*C*_i_ curve analysis

**Fig. 8:**
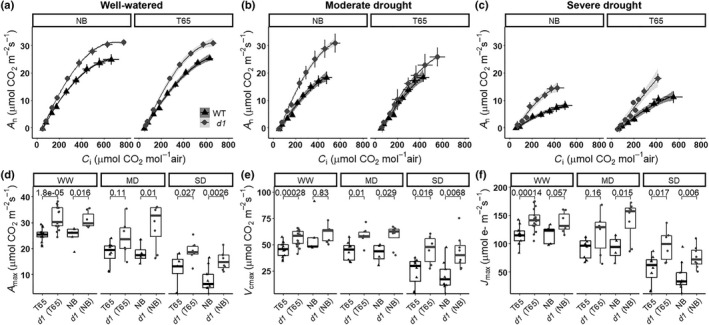
Rice *d1* mutants exhibit greater photosynthetic capacity relative to wild‐type (WT). (a–c) CO_2_ assimilation in response to changes in CO_2_ concentration in the leaf intercellular air spaces *C*_i_ in the different rice genotypes: WT ‘Taichung 65’ (T65) and ‘Nipponbare’ (NB), and *d1* in T65 background ‘*d1* (T65)’ and *d1* in NB background ‘*d1*(NB)’ under (a) well‐watered (WW) conditions (relative soil water content (RSWC) 100–50%), (b) moderate drought (MD) stress (RSWC 50–35%), and (c) severe drought (SD) stress (RSWC < 35%). Data are means ± SEs of WT (triangles) and *d1* (circles). Black lines represent the cubic curve fit for the data of the WT rice genotypes, and grey lines represent the cubic curve fit for the data of the *d1* mutant. The shaded areas show the 95% confidence interval of the regression lines. (d–f) Box plots of the physiological parameters calculated from the CO_2_ response curves of the different rice cultivars: WT ‘Taichung 65’ (T65) and ‘Nipponbare’ (NB), and *d1* in T65 background ‘*d1* (T65)’ and *d1* in NB background ‘*d1*(NB)’, under WW conditions (RSWC 100–50%), MD stress (RSWC 50–35%) and SD stress (RSWC < 35%), for (d) maximum photosynthetic rate *A*_max_, (e) maximum capacity for Rubisco carboxylation normalized to 25°C *V*_cmax_, and (f) maximum electron transport rate normalized to 25°C *J*_max_. The upper and lower parts of the boxes represent the 25^th^ and 75^th^ percentiles, respectively. The horizontal line within the boxes marks the median. The whiskers reach the largest and smallest values. Points are individual measurements. Connecting group lines indicate the *P*‐value score of the means between WT and *d1* according to Student's *t*‐test.

We also aimed to determine in these genotypes the contributions to the photosynthetic limitation of CO_2_ diffusion (stomatal and mesophyll conductance) vs the biochemical carboxylation capacity of the enzyme Rubisco (*V*_cmax_). As observed from *A*_n_ *–C*_i_ curves (Fig. 8a–c), *d1* also exhibited greater photosynthetic capacity: at saturating CO_2_, maximum CO_2_ assimilation *A*_max_ was greater in *d1*. Analysis of the *A*–*C*_c_ curves revealed that Rubisco capacity for carboxylation normalized to 25°C (*V*_cmax_), and electron transport capacity normalized to 25°C (*J*_max_) were significantly higher in *d1* than in WT (Fig. 8d–f) under both well‐watered and drought conditions for both T65 and NB backgrounds.

### RNA‐sequencing analysis

**Fig. 9:**
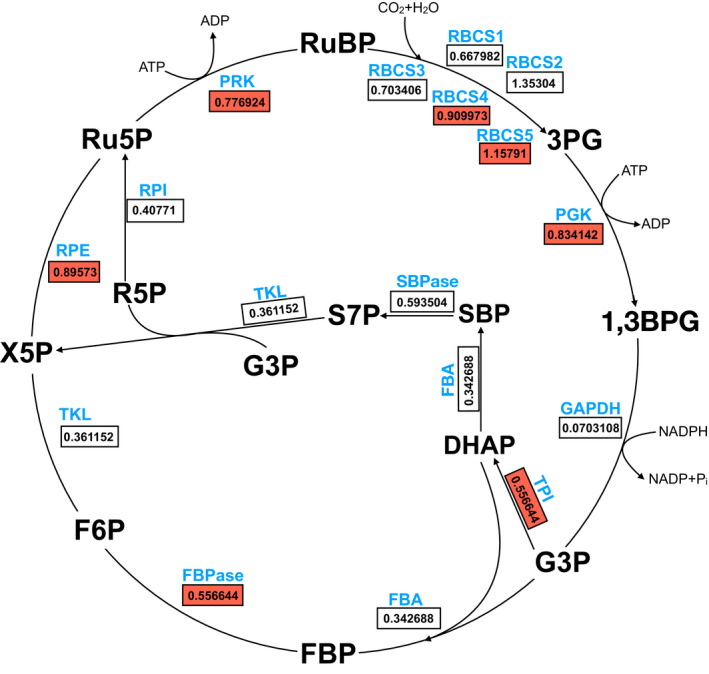
Rice *d1* mutant exhibits an upregulation in messenger RNAs (mRNAs) encoding key Calvin cycle enzymes. The Calvin cycle illustrating genotypic differences in the expression of photosynthesis‐related genes (Supporting Information Table S3). Statistically different expression levels between ‘Taichung 65’ and *d1* were designated as those where *q*‐values were less than 0.05 and fragments per kilobase of exon per million mapped fragments was greater than one in at least one genotype. Orange boxes represent genes exhibiting statistically significant upregulation of mRNA in *d1*. White boxes represent nonstatistically significant different regulation of RNA between *d1* and wild‐type (WT). Values inside orange and white boxes indicate the differential regulation (Log2 fold‐change) of mRNA in *d1* relative to WT. 1,3BPG, 1,3‐bisphosphoglycerate; 3PG, 3‐phosphoglycerate; DHAP, dihydroxyacetone phosphate; F6P, fructose 6‐phosphate; FBA, fructose‐bisphosphate aldolase; FBP, fructose 1,6 bisphosphate; FBPase, fructose‐1,6‐bisphosphatase; G3P, glyceraldehyde 3‐phosphate; GAPDH, glyceraldehyde 3‐phosphate dehydrogenase; PGK, phosphoglycerate kinase; PRK, phosphoribulokinase; R5P, ribose 5‐phosphate; RBCS, Rubisco small subunit; RPE, ribulose‐phosphate 3‐epimerase; RPI, ribose‐5‐phosphate isomerase; Ru5P, ribulose 5‐phosphate; RuBP, ribulose 1,5‐bisphosphate; S7P, sedoheptulose 7‐phosphate; SBP, sedoheptulose 1,7‐bisphosphate; SBPase, sedoheptulose 1,7 bisphosphatase; TKL, transketolase; TPI, triosephosphate isomerase; X5P, xylulose‐5‐phosphate.

We conducted a reanalysis of RNA‐seq data from T65 and *d1* seedlings under well‐watered conditions (Ferrero‐Serrano *et al*., 2018) to explore genotypic differences in the expression of genes implicated in the regulation and modulation of *g*_m_. Our analysis revealed differential expression of several plasma membrane intrinsic proteins (PIP) aquaporin genes (Table S1) and different alpha and beta CAs (Table S2). However, we did not find a consistent upregulation of these genes in one genotype vs the other that would infer at the transcriptional level a role of these gene families in the regulation of *g*_m_. However, our reanalysis of this RNA‐seq data set reveals a consistent upregulation in *d1* of key regulatory enzymes of the Calvin–Benson cycle (Fig. 9; Table S3).

## Discussion

### Rice *RGA1* mutant *d1* exhibits increased mesophyll conductance to CO_2_

Improving mesophyll conductance has the potential to boost photosynthesis and crop water use efficiency, especially under drought conditions. However, any goal to manipulate *g*_m_ for crop improvement first requires an understanding of its genetic and physiological basis. We discovered that the canonical α subunit of the rice heterotrimeric G protein, RGA1, is a regulator of mesophyll conductance. Our results confirm previously reported enhanced drought tolerance of *d1* plants in the T65 background (Ferrero‐Serrano & Assmann, 2016; Cui *et al*., 2020), but additionally show cultivar independence, as *d1* is also more drought tolerant in the NB background. Such cultivar independence is essential toward incorporating the dwarfing and drought tolerance phenotypes of *d1* into elite cultivars. Mechanistically, our results reveal that this drought tolerance is related to higher *g*_m_ in *d1*, which confers higher CO_2_ assimilation rates over the drought time course. The greater *g*_m_ in the *d1* mutants for both genotypic backgrounds (T65 and NB) allows greater CO_2_ concentration at the sites of carboxylation in the chloroplast (*C*_c_), leading to greater CO_2_ assimilation rates without any increase in stomatal conductance and transpiration, thus resulting in an increase in WUE_i_ under drought conditions (Figs 2, 3).

Under well‐watered conditions, *d1* displays higher *g*_s_, which leads to lower WUE_i_ (Fig. 3). Increasing WUE_i_ is a desirable trait, especially for plants growing under unfavorable conditions. However, in the absence of drought, higher *g*_sw_ is beneficial for increased CO_2_ supply for photosynthesis and for substantial transpiration, which can confer leaf cooling and thus improve heat tolerance (Gates, 1968), which is of particular concern due to global warming. Indeed, we previously observed lower leaf temperatures in *d1* than in WT, which may also be conferred by the more erect stature of *d1* as opposed to WT (Ferrero‐Serrano *et al*., 2018). However, despite its lower WUE_i_ under well‐watered conditions, *d1* mutants under drought increased WUE_i_ via stomatal closure while maintaining high *g*_m_ (Figs 2, 3). In other words, the dynamic response of *g*_s_ changes the relative importance of *g*_s_ and *g*_m_ during drought: *d1* exhibited a delayed decline in *g*_m_ relative to that of *g*_s_ (Fig. 4), which led to higher *A*_n_ under drought. A higher *g*_m_ : *g*_s_ ratio under limited water conditions has been previously documented for other highly drought‐tolerant species, such as eucalyptus, poplar, and *Ziziphus spina‐christi* trees (Cano *et al*., 2014; Théroux‐Rancourt *et al*., 2015; Zait *et al*., 2019) and could be a key trait for selection and breeding for WUE_i_ improvement in crops (Barbour *et al*., 2010; Barbour & Kaiser, 2016; Flexas *et al*., 2016; Tomeo & Rosenthal, 2017).

### Role of *RGA1* in mesophyll anatomy and diffusive conductance to CO_2_

Leaf anatomy is an important determinant of *g*_m_ from the substomatal cavities through the intercellular air space to the carbon fixation sites in the chloroplast (Nobel, 1991; Parkhurst, 1994). G protein signaling has been shown to play a crucial role in developmental processes in a number of plant species, including rice (Perfus‐Barbeoch *et al*., 2004; Urano *et al*., 2020). For instance, loss‐of‐function mutations in the Gα subunit of the heterotrimeric G protein mediate morphological changes in cell proliferation in Arabidopsis and maize (Wang *et al*., 2001; Urano *et al*., 2016), shoot meristem size in maize (Bommert *et al*., 2013), Arabidopsis internode length in Arabidopsis (Ueguchi‐Tanaka *et al*., 2000), and root morphology (Chen *et al*., 2006; Ding *et al*., 2008) in Arabidopsis.

Our results showed that *d1* mutants display altered mesophyll (*S*_m_) and chloroplast (*S*_c_) area exposed to intercellular airspaces, parameters that have previously been shown to positively correlate with the increase of *g*_m_, WUE_i_, and *A*_max_ in rice (Giuliani *et al*., 2013; Xiong *et al*., 2016; Ouyang *et al*., 2017). The WT values of the anatomical parameters obtained in this study are within the ranges described in the literature for rice (Giuliani *et al*., 2013; Xiong *et al*., 2015b, 2017; Ouyang *et al*., 2017; Ellsworth *et al*., 2018). The *d1* mutants were found to display reductions in cell wall thickness and chloroplast thickness (Fig. 6; Table 1), which together accounted for a reduction of 53% in the CO_2_ liquid‐phase resistance compared with WT (Fig. 7). Reduced cell wall thickness in the *d1* mutant is supported by the latest finding that RGA1 modulates genes involved in cell wall composition in rice (Pathak *et al*., 2021). Moreover, in Arabidopsis, G proteins have been shown to interact with proteins involved in cell wall modification (Klopffleisch *et al*., 2011) and to regulate trafficking of cellulose synthase to the plasma membrane (McFarlane *et al*., 2021). Indeed, differences in cell wall composition of G protein mutants have been identified by Fourier transform infrared spectroscopy (Delgado‐Cerezo *et al*., 2012).

The anatomical characteristics of the *d1* mutant leaf can explain the higher *g*_m_ in *d1* over the WT under well‐watered conditions. However, an interesting question is how leaf anatomy contributes to any changes in *g*_m_ and photosynthesis under drought. *g*_m_ is known to be affected by light transmittance through mesophyll layers (Théroux‐Rancourt & Gilbert, 2017) (Fig. S4). The erect architecture of the *d1* leaves reduces the amount of incident light intercepted by the leaves (Setter *et al*., 1996), which enables more efficient light capture and protects the photosynthesis machinery from photoinhibition (Ferrero‐Serrano *et al*., 2018). Moreover, drought stress exacerbates photoinhibition and increases the need for effective photoprotection (Cornic & Fresneau, 2002; Takahashi & Badger, 2011). We therefore suggest that *d1* mutants increase light use efficiency and photosynthesis by architectural avoidance of high light intensities, which redistributes chloroplasts for maximal light interception and, thus, reduces the diffusional pathlength from the cell wall to the chloroplast envelope, and by increased chloroplast surface area, both of which consequently increase *g*_m_.

### Role of metabolic factors in the regulation of mesophyll conductance in the *d1* mutant

Increases in leaf temperature occur alongside drought due to stomatal closure. We showed that *g*_m_ dynamically changes in response to leaf temperature (Fig. 5) and CO_2_ concentration (Fig. S5). Because these changes occur on a rapid timescale, they are unlikely to involve anatomical changes (Bernacchi *et al*., 2002; Carriquí *et al*., 2019). We accordingly hypothesize that metabolic processes could contribute to the differential reduction in *g*_m_ we observe between WT and *d1* mutants over the drought time course. The erect architecture of *d1*, together with its higher values of *g*_sw_ under well‐watered conditions (Fig. 2), reduces leaf temperature compared with WT (Ferrero‐Serrano & Assmann, 2016; Ferrero‐Serrano *et al*., 2018). Our results suggest that, at extreme ambient temperatures, *d1* architecture could help maintain the leaves at the optimal temperature for maximum *g*_m_ (Fig. 5c). Changes in leaf temperature can rapidly affect *g*_m_, especially in crops typical of warm and tropical habitats, such as rice (von Caemmerer & Evans, 2015). Though the mechanism is yet to be determined, previous studies have suggested that temperature exerts a direct effect on membrane permeability to CO_2_ by activating and increasing expression of aquaporins (Bernacchi *et al*., 2002; Groszmann *et al*., 2017; Qiu *et al*., 2017).

It has been previously shown that overexpression of the aquaporin PIP2 increased *g*_m_ by 40% in transgenic rice plants, highlighting the role of aquaporins in the diffusion of CO_2_ through the mesophyll cell membrane (Hanba *et al*., 2004). This contrasts with the lack of changes in *g*_m_ observed in Arabidopsis aquaporin *PIP1* and *PIP2* knockout lines relative to Col‐0 (Kromdijk *et al*., 2020). In cucumber, suppression of the Gα subunit results in the overexpression of several aquaporins under well‐watered conditions (Yan *et al*., 2020). Our RNA‐seq analysis shows that some *PIP* aquaporin genes are differentially expressed in *d1* mutants (Table S1), but these do not exhibit a consistent response: *PIP1.3*, *PIP2.4*, and *PIP2.1* are downregulated and *PIP2‐7* is upregulated in *d1*. It is important to note that leaves sampled for the RNA‐seq reanalysis that we report here were harvested under well‐watered conditions. Thus, it remains possible that aquaporins actively change expression or activity during drought (Huang *et al*., 2021), with consequent impacts on *g*_m_. In rice, *OsPIP1.1* and *OsPIP2.1* were found to be upregulated in both roots and leaves in response to water depletion (Guo *et al*., 2006). Further studies on rice aquaporins, including their CO_2_ permeability and whether their expression and activity are drought regulated, are needed to assess their relative importance to the metabolic component of *g*_m_ in WT vs *d1*.

Another factor affecting resistance along the CO_2_ diffusion pathways is CA (Flexas *et al*., 2012). CA catalyzes the interconversion of CO_2_ and HCO_3_^−^ (Momayyezi *et al*., 2020). In rice, the downregulation of CA activity by a deficiency in zinc (a co‐factor of CA) results in a 2.3‐fold reduction in *g*_m_, emphasizing the importance of CA (Sasaki *et al*., 1998). Our RNA‐seq analysis under well‐watered conditions shows upregulation in *d1* relative to WT of the genes encoding the chloroplast‐abundant αCA1 (Table S2) and the βCA1 (Table S2). However, CA enzyme activity assays show no statistically significant differences between the *d1* mutant and WT (Fig. S6).

It is important to point out that, in Arabidopsis, the G protein α subunit knockout mutant, *gpa1‐3*, can accumulate more biomass, with less water loss (Nilson & Assmann, 2010b). The *gpa1‐3* mutant exhibits reduced discrimination to ^13^C in the absence and presence of ABA, which strongly correlates with high water use efficiency (Nilson & Assmann, 2010b). The *gpa1‐3* mutant is hyposensitive to ABA inhibition of stomatal opening (Wang *et al*., 2001), which could negatively affect WUE_i_ under drought. However, recent evidence shows that ABA has an essential role in the regulation of *g*_m_. Higher exogenous ABA in the leaves decreases *g*_m_ (Mizokami *et al*., 2015), presumably by reducing the aquaporins’ plasma membrane CO_2_ conductivity (Shatil‐Cohen *et al*., 2011; Sorrentino *et al*., 2016). Thus, a higher *g*_m_ in *d1* in the presence of drought may arise from lower sensitivity to ABA in the mesophyll cells. Accordingly, the role of RGA1 in specific tissues requires further investigation.

### Improved photosynthetic capacity in the *d1* mutant

Our results show that *d1* exhibits a higher maximum capacity for Rubisco carboxylation *V*_cmax_, maximum electron transport rate *J*_max_, and maximum CO_2_ assimilation *A*_max_ relative to WT (Fig. 8). Photosynthetic capacity is related to the nitrogen (N) content because the proteins of the Calvin cycle and thylakoids contain the majority of leaf N (Evans, 1989). The majority of N in the thylakoids is found in the photosystem reaction centers (Evans, 1989). As shown in a previous study, photosynthetic reaction center genes are upregulated in *d1* plants (Ferrero‐Serrano *et al*., 2018). Our reanalysis of the RNA‐seq data from that study also reveals the upregulation in *d1* of key enzymes of the Calvin–Benson cycle (Fig. 9; Table S3). We hypothesize that the upregulation of the biochemical factors of photosynthesis in the *d1* mutant may arise in part from allocation of a larger fraction of leaf N to photosynthesis processes, resulting in an increase of photosynthetic N use efficiency (PNUE) (Gao *et al*., 2020). This hypothesis is also supported by the typical dark‐green leaf phenotype (Fujisawa *et al*., 1999), higher Chl content, and upregulation of key enzymes in Chl biosynthesis observed in the *d1* mutant (Ferrero‐Serrano *et al*., 2018). A larger PNUE in rice is associated with a higher *S*_c_ and thinner cell walls, leading to a higher *g*_m_ (Li *et al*., 2009; Gao *et al*., 2020; Ye *et al*., 2020). Additionally, we observed that *g*_m_ was more sensitive to elevated CO_2_ in *d1* relative to WT, under both well‐watered and drought conditions (Fig. S5). This is consistent with the finding that N deficiency in rice leads to hyposensitivity of *g*_m_ to changes in light and CO_2_ (Xiong *et al*., 2015).

### Conclusion

The rice G protein Gα subunit, RGA1, regulates mesophyll conductance in large part by altering mesophyll anatomy. This is the first identification of a gene that pleiotropically affects multiple aspects of mesophyll anatomy related to *g*_m_. In future research, it will be important to determine the cell signaling mechanisms whereby RGA1 controls such disparate anatomical properties as cell wall thickness, mesophyll cell size, and chloroplast surface area. Moreover, the *d1* mutants also have elevated photosynthetic capacity compared with the WT congeners. Accordingly, the results of this study demonstrate that genetic modulation of heterotrimeric G protein activity in rice provides a novel strategy to increase *g*_m_ and WUE_i_ under drought. In rice, the introduction of dwarfism was a crucial agronomic advance (Hargrove & Cabanilla, 1979; Hedden, 2003; Ferrero‐Serrano *et al*., 2019), but it currently has a very narrow genetic basis (Chang & Vergara, 1972; Hargrove *et al*., 1980). Current elite varieties display drought sensitivity, which is associated with introgression of the chromosomal region containing the Green Revolution dwarfing gene *sd1* (Vikram *et al*., 2015). We propose that the manipulation of G protein signaling in rice offers a novel strategy not only to confer the desired semi‐dwarf phenotype but also to improve photosynthesis and water use efficiency and increase drought tolerance.

## Author contributions

YZ planned and performed the experiments, analyzed the data, and wrote the text; AF‐S performed preliminary experiments, analyzed the RNA‐seq data (Fig. 9; supporting information tables) and wrote the text. SMA planned and designed the research and wrote the text.

## Supporting information


**Fig. S1** Estimation of mitochondrial respiration in the light (*R*_d_) and the intercellular CO_2_ compensation point ($C_{i}^{*}$) in WT rice and the *d1* mutant.
**Fig. S2** Different estimation methods for *g*_m_ all maintain the difference in *g*_m_ observed between WT rice and *d1* mutants.
**Fig. S3** Relationship between photochemical efficiency of PSII (ΦPSII) and apparent quantum yield of CO_2_ assimilation (ΦCO_2_) under non‐photorespiratory conditions (1% O_2_).
**Fig. S4** Light responses of CO_2_ assimilation rate (*A*_n_) (a), mesophyll conductance (*g*_m_) (b), and stomatal conductance to water vapor (*g*_sw_) (c), in wild‐type rice Taichung 65 and the *d1* mutant.
**Fig. S5** The rapid response of mesophyll conductance (*g*_m_) to changes in CO_2_ concentration in the leaf intercellular air spaces (*C*_i_) in different rice genotypes.
**Fig. S6** WT rice and the *d1* mutant do not differ in carbonic anhydrase activity.
**Table S1** Expression levels of plasma membrane intrinsic proteins (PIPs) in Taichung 65 wild‐type rice (WT) vs. the Taichung 65 *d1* mutant.
**Table S2** Expression levels of carbonic anhydrases in Taichung 65 wild‐type rice (WT) vs the Taichung 65 *d1* mutant.
**Table S3** Expression levels of Calvin cycle enzymes in Taichung 65 wild‐type rice (WT) vs the Taichung 65 *d1* mutant.
Please note: Wiley Blackwell are not responsible for the content or functionality of any Supporting Information supplied by the authors. Any queries (other than missing material) should be directed to the *New Phytologist* Central Office.

## Data Availability

All TEM images have been uploaded to GitHub and are publicly accessible at https://github.com/AssmannLab/rice_mesophyll_conductance. All other data are available upon request from the authors.
