# Supplementary material for: The α subunit of the heterotrimeric G protein regulates mesophyll CO2 conductance and drought tolerance in rice
Source: New Phytol. 2021 Sep 30;232(6):2324–38. doi: 10.1111/nph.17730 (PMC9293471; doi:10.1111/nph.17730)
Supplement: Supplementary file 1 — Fig. S1 Estimation of mitochondrial respiration in the light (R d) and the intercellular CO2 compensation point (Ci∗) in WT rice and the d1 mutant. Fig. S2 Different estimation methods for g m all maintain the difference in g m observed between WT rice and d1 mutants. Fig. S3 Relationship between photochemical efficiency of PSII (ΦPSII) and apparent quantum yield of CO2 assimilation (ΦCO2) under non‐photorespiratory conditions (1% O2). Fig. S4 Light responses of CO2 assimilation rate (A n) (a), mesophyll conductance (g m) (b), and stomatal conductance to water vapor (g sw) (c), in wild‐type rice Taichung 65 and the d1 mutant. Fig. S5 The rapid response of mesophyll conductance (g m) to changes in CO2 concentration in the leaf intercellular air spaces (C i) in different rice genotypes. Fig. S6 WT rice and the d1 mutant do not differ in carbonic anhydrase activity. Table S1 Expression levels of plasma membrane intrinsic proteins (PIPs) in Taichung 65 wild‐type rice (WT) vs. the Taichung 65 d1 mutant. Table S2 Expression levels of carbonic anhydrases in Taichung 65 wild‐type rice (WT) vs the Taichung 65 d1 mutant. Table S3 Expression levels of Calvin cycle enzymes in Taichung 65 wild‐type rice (WT) vs the Taichung 65 d1 mutant. Please note: Wiley Blackwell are not responsible for the content or functionality of any Supporting Information supplied by the authors. Any queries (other than missing material) should be directed to the New Phytologist Central Office. [file NPH-232-2324-s001.pdf]

New Phytologist Supporting Information

The α subunit of the heterotrimeric G protein regulates mesophyll CO_2 conductance and drought tolerance in rice.

Yotam Zait, Ángel Ferrero-Serrano, Sarah M. Assmann

Acceptance date: 1 September 2021

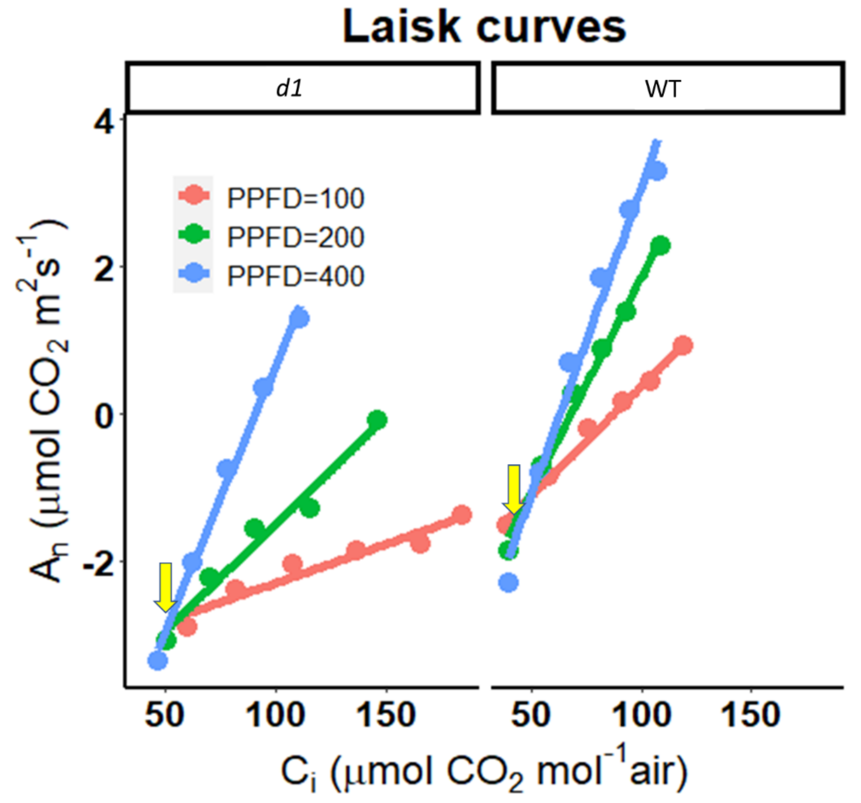

Figure S1: Estimation of mitochondrial respiration in the light (Rd) and the intercellular CO_2 compensation point (Ci*) in WT rice and the *d1* mutant. Estimation of the chloroplast CO_2 compensation point (Γ^*) as the intercept of the regression between mitochondrial respiration in the light (R_d) and the intercellular CO_2 compensation point (Ci*) under 3 different light intensities: 400, 200 and 100 µmol m^{-2} s^{-1} at CO_2 concentrations of 200, 150, 100, 75, 50 and 25 µmol mol^{-1}. Arrows represents example calculation of R_d and Ci* using the "Laisk method" for WT (Rd=1.6 µmol m^{-2} s^{-1}, C_i*=47 µmol mol^{-1}) and for the *d1* mutant (R_d=2.2 µmol m^{-2} s^{-1}, C_i*=48 µmol mol^{-1}).

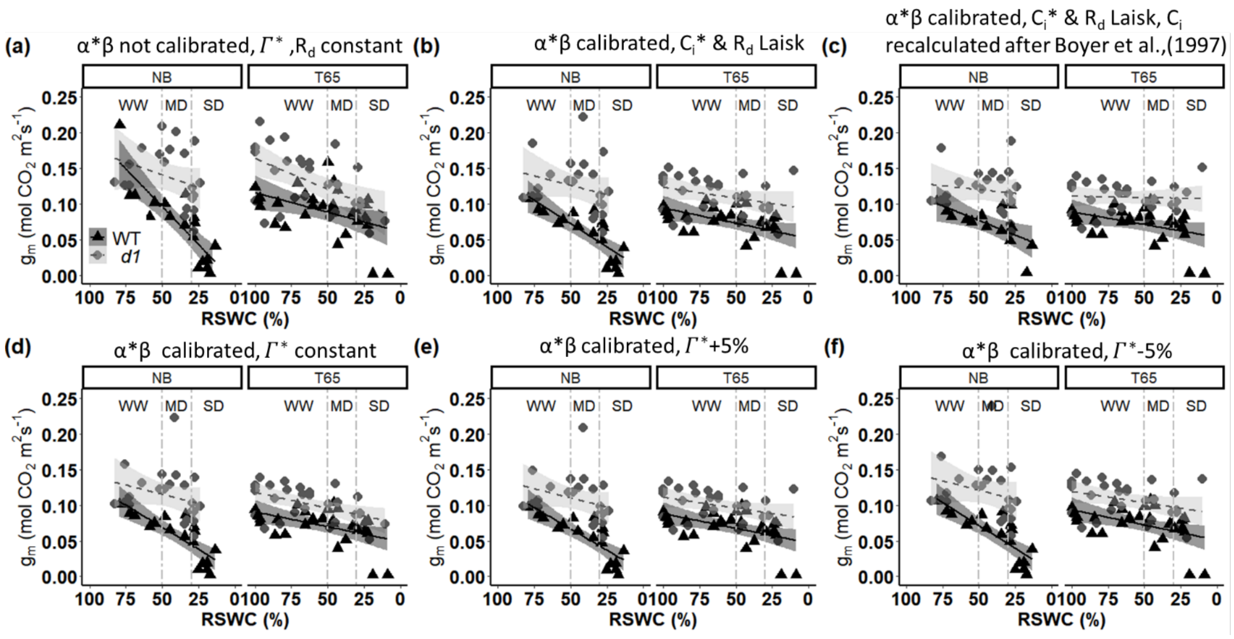

Figure S2: Different estimation methods for g_m all maintain the difference in g_m observed between WT rice and *d1* mutants. Relationships between mesophyll conductance to CO_2 (g_m) and the decline in relative soil water content (RSWC) for different g_m estimation methods when: (a) αβ not calibrated (0.842), with constant Γ* (38.5 µmol mol^{-1}) and R_d (1.5µmol m^{-2} s^{-1}), (b) αβ calibrated after Valentini *et al.*, (1995) and Ci* and R_d calculated from Laisk curves, (c) αβ calibrated after Valentini et al., (1995) and Ci* and R_d calculated from Laisk curves and Ci corrected after Boyer *et al.*, (1997), (d) αβ calibrated after Valentini et al., with constant Γ* (38.5 µmol mol^{-1}) and constant R_d (1.5µmol m^{-2} s^{-1}), (e) αβ calibrated after Valentini et al. (1995), Γ* was taken Bernacchi *et al.*, (2002) and increased by 5% with drought severity or (f) decreased by 5% with drought severity. Continuous lines represent the linear fit for the data of the WT rice genotypes, and dashed lines represent the linear fit for the data of the *d1* mutants. The shaded areas show the 95% confidence interval of the regression lines.

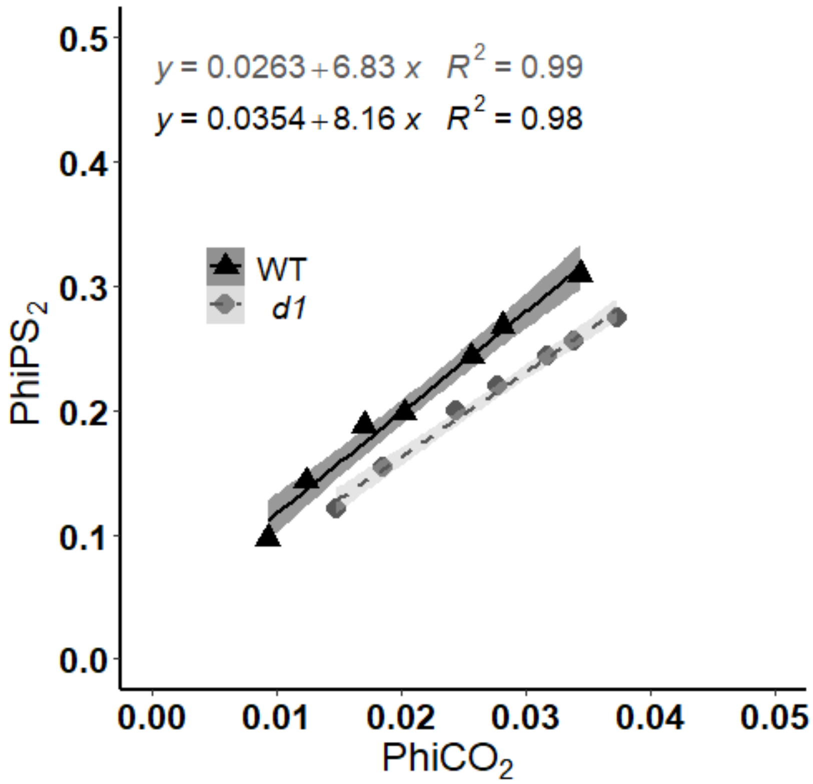

Figure S3: Relationship between photochemical efficiency of PSII (ΦPSII) and apparent quantum yield of CO_2 assimilation (ΦCO_2) under non-photorespiratory conditions (1% O_2). Continuous lines represent the linear fit for the data of the WT rice genotype, and dashed lines represent the linear fit for the data of the *d1* mutant. The shaded areas show the 95% confidence interval of the regression lines.

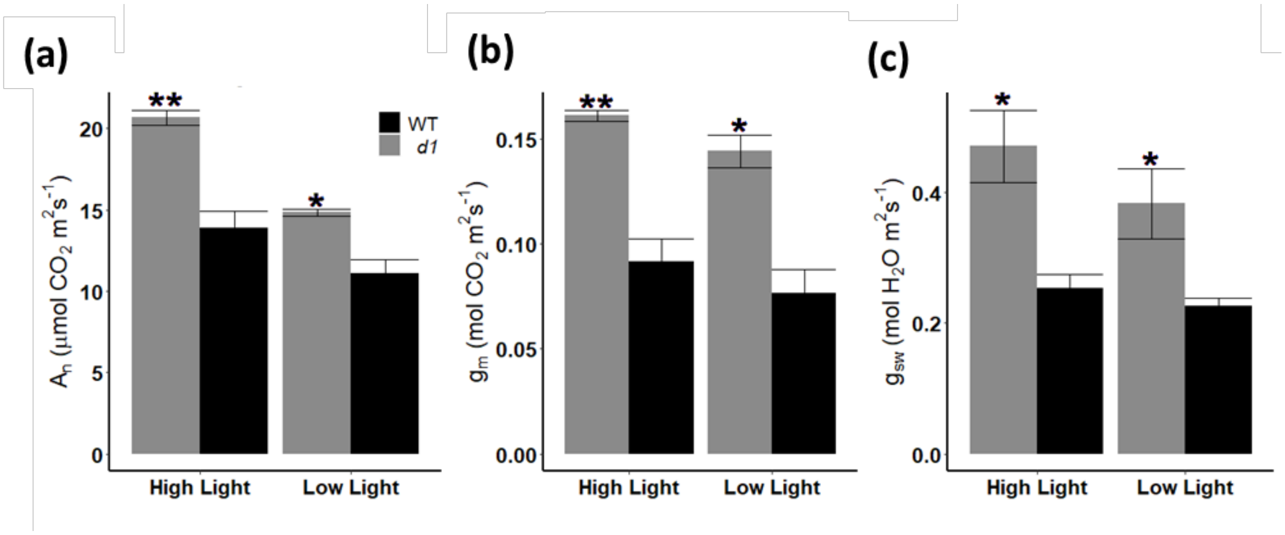

Figure S4: Light responses of CO_2 assimilation rate (A_n) (a), mesophyll conductance (g_m) (b), and stomatal conductance to water vapor (g_{sw}) (c), in wild-type rice Taichung 65 and the *d1* mutant. Plants were initially measured at low light intensity (PPFD=500 µmol m^{-2} s^{-1}) before increasing to high light intensity (1500 µmol m^{-2} s^{-1}). Error bars are S.E. Asterisks indicate that means (n=5) differ significantly from the mean of the corresponding WT as: P<0.05 (*), P<0.001(**), according to Student's t-test.

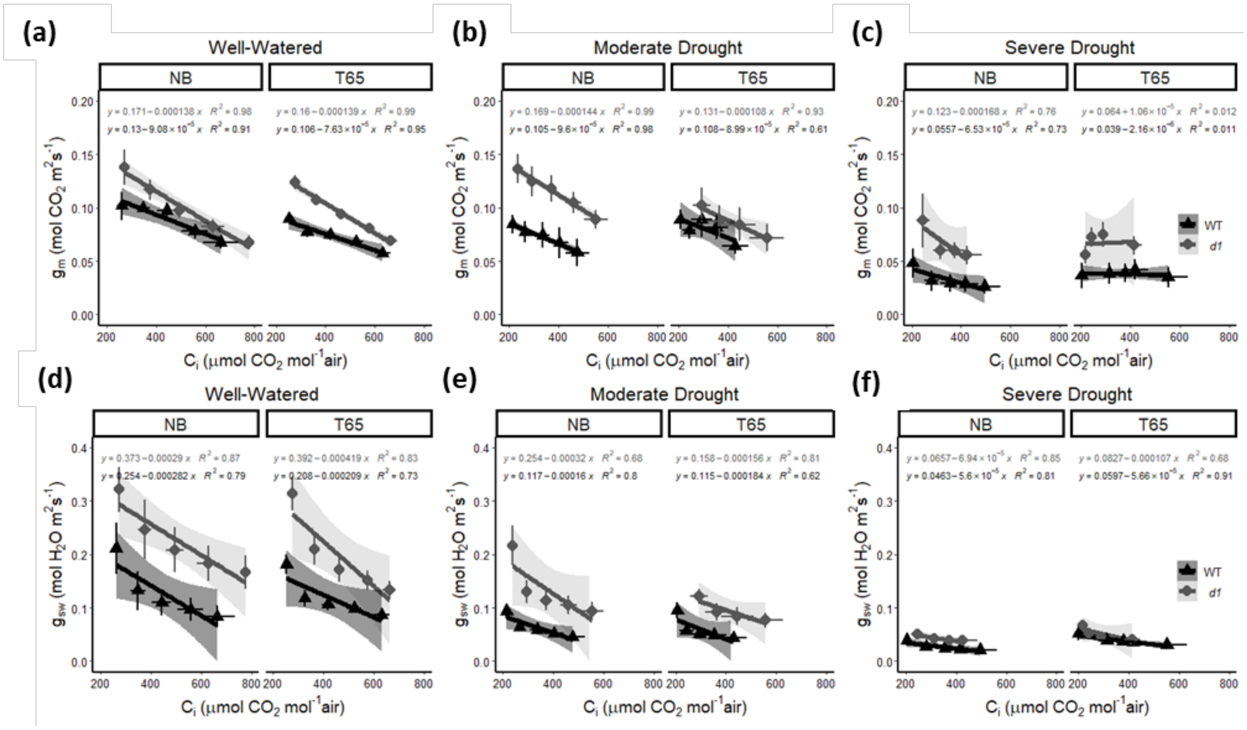

Figure S5: The rapid response of mesophyll conductance (g_m) to changes in CO_2 concentration in the leaf intercellular air spaces (C_i) in different rice genotypes. Data are for wild-type Taichung 65 (T65) and Nipponbare (NB), and *d1* in T65 background "*d1* (T65)" and *d1* in Nipponbare background "*d1*(NB)" under: (A) well-watered conditions (RSWC 100-50%), (B) moderate drought stress (RSWC 50-35%), and (C) severe drought stress (RSWC <35%), and the response of the stomatal conductance to water vapor (g_{sw}) to C_i for the different rice genotypes under (D) well-watered conditions (RSWC 100-50%), (E) moderate drought stress (RSWC 50-35%) and (F) severe drought stress (RSWC <35%). Data are means ± standard errors of WT (triangles) and *d1* (circles). Continuous lines represent the linear fit for the data of the WT rice genotypes, and dashed lines represent the linear fit for the data of the *d1* mutants. The shaded areas show the 95% confidence interval of the regression lines.

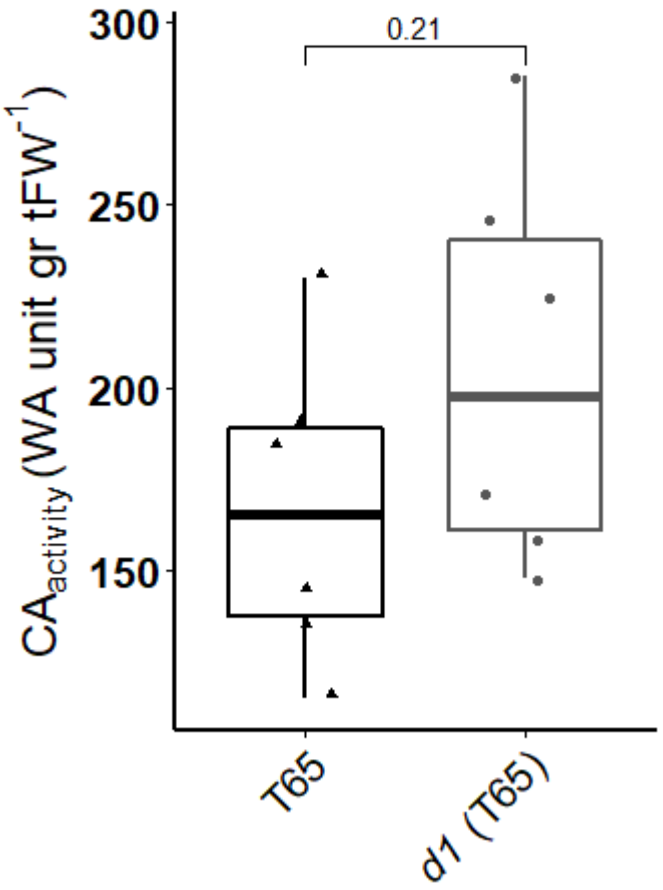

Figure S6: WT rice and the *d1* mutant do not differ in carbonic anhydrase activity. Catalytic carbonic anhydrase activity in the wild-type Taichung 65 (T65) and corresponding *d1* mutant (n=6). The upper and lower part of the boxes represent the 25th and 75th percentiles. The horizontal line within the boxes marks the median. The whiskers reach the largest and smallest values. Points are individual measurements of WT (triangles) and *d1* (circles). Connecting group line indicate the P value score of the means between WT and *d1* according to Student's t-test.

Table S1. Expression levels of plasma membrane intrinsic proteins (PIPs) in Taichung 65 wild-type rice (WT) vs. the Taichung 65 *d1* mutant. Upregulated transcripts are highlighted in bold and underlined font.

Locus ID	WT	*d1*	q-value	Gene ID	Description
OS02G0666200	1266.75	**1403.12**	0.65909	PIP1.1	Plasma membrane intrinsic protein 1.1
OS04G0559700	435.755	**448.89**	0.91259	PIP1.2	Plasma membrane intrinsic protein 1.2
Os02G0823100	**380.298**	115.185	0.0001863	PIP1.3	Plasma membrane intrinsic protein 1.3
Os07G0448800	**427.41**	287.51	0.0080311	PIP2A	Plasma membrane intrinsic protein 2.1
Os02G0629200	**434.651**	341.73	0.322603	PIP2.2	Plasma membrane intrinsic protein 2.2
OS04G0521100	0	0	1	PIP2.3	Plasma membrane intrinsic protein 2.3
OS07G0448100	**15.5936**	1.98629	0.0001863	PIP2.4	Plasma membrane intrinsic protein 2.4
OS07G0448400	**0.878948**	0.782262	0.926896	PIP2.5	Plasma membrane intrinsic protein 2.5
OS04G0233400	12.5667	**15.7035**	0.368986	PIP2.6	Plasma membrane intrinsic protein 2.6
OS09G0541000	213.722	**357.907**	0.0086542	PIP2.7	Plasma membrane intrinsic protein 2.7
Os03G0861300	0	0	1	PIP2.8	Plasma membrane intrinsic protein 2.8
Os07G0448200	0.133641	0	1	PIP2.9	Plasma membrane intrinsic protein 2.9
OS10G0481100	0	0	1	PIP2.10	Plasma membrane intrinsic protein 2.10

Table S2. Expression levels of carbonic anhydrases in Taichung 65 wild-type rice (WT) vs. the Taichung 65 *d1* mutant. Upregulated transcripts are highlighted in bold and underlined font.

Locus ID	WT	*d1*	Log2 Fold-change	q-value	Gene ID	Description
**OS02G0533300**	8.83911	**13.453**	0.605952	0.0237823	ALPHACA1	Alpha-carbonic anhydrase 1
**OS04G0412500**	93.4346	**116.477**	0.318017	0.162475	ALPHACA2	Alpha-carbonic anhydrase 2
**OS08G0423500**	0	0	0	1	ALPHACA3	Alpha-carbonic anhydrase 3
**OS08G0423600**	0	0	0	1	ALPHACA4	Alpha-carbonic anhydrase 4
**OS08G0424100**	0	0	0	1	ALPHACA5	Alpha-carbonic anhydrase 5
**Os08G0470200**	**3.86159**	2.48383	-0.63663	0.161916	ALPHACA6	Alpha-carbonic anhydrase 6
**OS08G0470700**	**2.49483**	1.55259	-0.684262	0.238298	ALPHACA7	Alpha-carbonic anhydrase 7
**OS09G0454500**	0	0	0	1	ALPHACA9	Alpha-carbonic anhydrase 9
**OS11G0153200**	0	0	0	1	ALPHACA10	Alpha-carbonic anhydrase 10
**Os12G0153500**	0	0	0	1	ALPHACA11	Alpha-carbonic anhydrase 11
**OS01G0639900**	7339.38	**16887.5**	1.20223	0.0068879	BETACA1	Beta- carbonic anhydrase 1
**OS09G0464000**	**31.6521**	24.1406	-0.390838	0.252513	BETACA2	Beta- carbonic anhydrase 2

Table S3. Expression levels of Calvin cycle enzymes in Taichung 65 wild-type rice (WT) vs. the Taichung 65 *d1* mutant. Upregulated transcripts are highlighted in bold and underlined font.

**Locus ID**	**WT**	***d1***	**Log2 Fold-change**	**q-value**	**Gene ID**	**description**
OS02G0152400	1.41054	**2.24113**	0.667982	0.243373	RBCS1	RuBisCO small subunit 1
OS12G0274700	6176.39	**15777.6**	1.35304	0.0800543	RBCS2	RuBisCO small subunit 2
OS12G0291100	21643.7	**35243.3**	0.703406	0.255504	RBCS3	RuBisCO small subunit 3
OS12G0292400	1756.85	**3301.14**	0.909973	0.0160891	RBCS4	RuBisCO small subunit 4
OS12G0291400	1430.35	**3191.62**	1.15791	0.0362096	RBCS5	RuBisCO small subunit 5
OS05G0496200	2049.22	**3653.35**	0.834142	0.0101391	PGK	Phosphoglycerate kinase
OS06G0136600	108.698	**114.127**	0.0703108	0.80668	GAPDH	Glyceraldehyde-3-phosphate dehydrogenase
OS01G0866400	318.398	**468.314**	0.556644	0.0202096	FBPase	Fructose biphosphatase
Os06G0133800	1128.95	**1450.08**	0.361152	0.28974	TKL	Transketolase
OS03G0169100	913.92	**1700.39**	0.89573	0.0030396	RPE	Ribulose-5-phosphate epimerase
OS02G0698000	1993.38	**3415.6**	0.776924	0.0187343	PRK	Phosphoribulokinase
Os09G0535000	631.809	**1018.51**	0.688908	0.0152739	TPI	Triosephosphatase isomerase
Os11G0171300	8812.96	**11175.9**	0.342688	0.614525	FBA	Fructose-biphosphate aldolase
OS04G0234600	1775.3	**2678.77**	0.593504	0.0637591	SBP	Sedoheptulose-1,7-biphosphatase
Os07G0176900	647.224	**858.593**	0.40771	0.103096	RPI	Ribulose-5-phosphate isomerase

References:

Bernacchi CJ, Portis AR, Nakano H, Caemmerer S Von, Long SP. **2002**. Temperature Response of Mesophyll Conductance. Implications for the Determination of Rubisco Enzyme Kinetics and for Limitations to Photosynthesis *in vivo*. *Plant Physiology* **130**: 1992–1998.

Boyer JS, Wong SC, Farquhar GD. **1997**. CO_2 and Water Vapor Exchange across Leaf Cuticle (Epidermis) at Various Water Potentials. *Plant Physiology* **114**: 185–191.

Valentini R, Epron D, Deangelis P, Matteucci G, Dreyer E. **1995**. *In situ* Estimation of Net CO_2 Assimilation, Photosynthetic Electron Flow and Photorespiration in Turkey Oak (*Q. cerris* L.) Leaves: Diurnal Cycles under Different Levels of Water-Supply. *Plant Cell & Environment* **18**: 631–640.
